# Incidence of Heterotopic Ossification Following Double-Incision Technique for Distal Biceps Tendon Repair

**DOI:** 10.7759/cureus.112453

**Published:** 2026-07-11

**Authors:** Seiji Nakahashi, Armando Secundino, Luis Arthur C Colferai, Filipe Baracho, Bernardo P Hespanhol, Guilherme Ferreira Gonzalez, Uheyna Gancedo Ruzon

**Affiliations:** 1 Department of Orthopaedics and Traumatology, Hospital do Trabalhador, Curitiba, BRA; 2 Department of Shoulder and Elbow Surgery, Hospital do Trabalhador, Curitiba, BRA

**Keywords:** distal biceps tendon rupture, double-incision technique, elbow surgery, heterotopic ossification, tendon repair

## Abstract

Background

Heterotopic ossification (HO) is a postoperative finding that may occur after distal biceps tendon repair using the double-incision technique; however, its incidence, temporal evolution, and clinical relevance remain incompletely defined.

Objectives

The objectives of this study are to determine the incidence and temporal evolution of heterotopic ossification (HO), evaluate variables associated with its development, and assess clinical outcomes following double-incision distal biceps tendon repair performed under a standardized postoperative protocol including non-steroidal anti-inflammatory drug (NSAID) prophylaxis.

Methods

A retrospective cohort study was performed including 94 patients with complete distal biceps tendon rupture treated surgically with a double-incision technique between January 2018 and July 2023. All patients had a minimum follow-up of 24 weeks and underwent serial radiographic evaluation at two, four, eight, 12, and 24 weeks postoperatively. HO was identified radiographically, and patients were grouped according to its presence. Functional outcomes were assessed using the Mayo Elbow Performance Score (MEPS). Clinical outcomes, need for additional surgery, and associated risk factors were analyzed.

Results

HO developed in 11 patients (11.7%), with spontaneous resorption observed in 54.5% of cases. Patients without HO achieved a mean MEPS of 97.8±2.5, whereas those with spontaneous HO resorption achieved a mean MEPS of 94.2±2.0. Persistent HO treated nonoperatively demonstrated a mean MEPS of 83.3±2.9, while surgically treated patients achieved a postoperative mean MEPS of 90.0±0. Only two patients (2.1%) required surgical excision. Anabolic steroid use and a delay greater than 10 days between injury and surgery were significantly associated with HO development (p<0.001).

Conclusion

The double-incision technique is associated with the development of HO; however, most cases follow a favorable and self-limited course with frequent spontaneous resorption and preservation of functional outcomes. Persistent symptomatic HO may result in functional limitation and occasionally require surgical intervention.

## Introduction

The incidence of distal biceps tendon rupture (DBTR) is estimated at approximately 5.35 cases per 100,000 individuals per year [1,2]. The majority of cases occur in the dominant limb of male patients between 30 and 50 years of age. In women, DBTR is rare and, when present, typically follows an insidious and atraumatic course, with partial tears accounting for most cases [3].

The classic mechanism of injury involves an eccentric contraction of the biceps during elbow extension with the forearm in supination. Two main theories have been proposed to explain the pathophysiology of DBTR: the presence of a hypovascular zone in the distal portion of the tendon and a reduction in the proximal radioulnar space during forearm rotation, leading to mechanical impingement of the tendon [2,4]. Non-simultaneous bilateral ruptures may occur in up to 8% of patients, suggesting that systemic risk factors play an important role in the development of this condition [5]. The most commonly reported risk factors include anabolic steroid use, smoking, obesity, and diabetes mellitus [6].

Nonoperative treatment has been associated with suboptimal functional outcomes, including persistent pain, fatigue, and a reduction in supination strength ranging from 21% to 55%, as well as decreased elbow flexion strength of 10% to 40%. In contrast, early surgical repair is associated with improved functional recovery and symptom relief [7,8].

Various surgical techniques have been described for DBTR repair, including single-incision and double-incision approaches, along with different methods of tendon fixation to bone. The single-incision technique has been associated with a higher incidence of nerve injuries, whereas the double-incision approach has been more frequently linked to the development of heterotopic ossification (HO) [8,9].

Although HO is a well-recognized complication, there remains a lack of robust data regarding its clinical course, temporal evolution, and optimal management following DBTR repair [10]. A better understanding of its natural history is essential to optimize postoperative surveillance and improve patient outcomes.

Therefore, the aim of this study was to evaluate the incidence and progression of heterotopic ossification in patients undergoing acute distal biceps tendon repair using a double-incision approach with prophylactic use of nonsteroidal anti-inflammatory drugs (NSAIDs). Additionally, this study sought to identify factors associated with the development and prognosis of HO, including its potential for spontaneous resorption over time.

## Materials and methods

This study was performed on a retrospective cohort based on a review of medical records of patients with complete distal biceps tendon rupture who underwent surgical treatment using the double-incision technique described by Boyd and Anderson [3] between January 2018 and July 2023.

The inclusion criteria were the following: (1) complete distal biceps tendon rupture confirmed clinically and/or by imaging studies; (2) primary surgical repair using the double-incision technique; (3) minimum postoperative follow-up of six months; and (4) availability of serial elbow radiographs in anteroposterior and lateral views obtained during the postoperative period (two, four, eight, 12, and 24 weeks).

The exclusion criteria were as follows: (1) patients treated nonoperatively; (2) patients undergoing alternative surgical techniques; (3) previous surgery or pre-existing sequelae involving the affected elbow; (4) incomplete medical records or missing radiographic data; and (5) loss to follow-up before the minimum six-month evaluation.

Functional outcomes were assessed at final follow-up using the Mayo Elbow Performance Score (MEPS), a validated instrument commonly used for the clinical evaluation of elbow disorders. The MEPS evaluates pain, range of motion, stability, and activities of daily living on a 100-point scale. Scores were categorized as excellent (90-100), good (75-89), fair (60-74), or poor (<60) [11]. Functional outcomes were assessed according to the presence and clinical evolution of heterotopic ossification.

Through an anterior incision at the antecubital fossa, the ruptured tendon was identified and prepared using two high-strength sutures in a modified Krakow configuration. A lateral approach through the Kocher approach was then performed to expose the radial tuberosity, where a bone trough measuring approximately 1 cm×0.5 cm was created. The tendon was delivered through the lateral incision, inserted into the bone trough, and secured using transosseous sutures through three 2-mm drill holes placed perpendicular to the radial tuberosity.

Postoperatively, prophylaxis for heterotopic ossification was administered with Celecoxib at a dose of 200 mg twice daily for 14 days.

Patients were immobilized in a sling for 30 days, with passive and active-assisted range of motion initiated after seven days, isometric exercises after 30 days, and progressive strengthening after 90 days. Clinical and radiographic evaluations were performed at two, four, eight, 12, and 24 weeks postoperatively. The presence of heterotopic ossification was determined based on serial radiographic assessment, and patients were categorized according to the presence or absence of this condition during follow-up.

Statistical analysis was performed using IBM SPSS Statistics version 29.0 (IBM Corp., Armonk, NY, USA). Normality of continuous variables was assessed using the Kolmogorov-Smirnov test. Comparisons between patients with and without heterotopic ossification were performed using the independent samples t-test or the Mann-Whitney U test, as appropriate, according to data distribution. Associations between categorical variables and the presence of heterotopic ossification were evaluated using Fisher’s exact test. Statistical significance was set at p<0.05. MEPS outcomes were analyzed descriptively according to HO status and treatment strategy, and no formal statistical comparisons between HO subgroups were performed because of the limited sample size within these groups.

## Results

A total of 102 medical records were reviewed, of which 94 patients met the inclusion criteria and were included in the study, while eight were excluded due to inadequate follow-up. The mean age at the time of surgery was 35.8±8.1 years. Most patients were men and younger than 45 years (83%), with the dominant limb affected in 71.3% of the cases.

The mean time from injury to surgical treatment was 7.4±3.1 days. During follow-up, 11 (11.7%) of 94 patients developed heterotopic ossification, whereas 83 (88.3%) did not. Demographic characteristics, risk factors, and clinical outcomes according to heterotopic ossification status are summarized in Table 1. No cases were observed within the first two weeks postoperatively; eight patients (8.5%) developed ossification at four weeks, two patients (2.1%) at 12 weeks, and one patient (1.1%) presented ossification only at the 24-week radiographic evaluation (Table 2).

**Table 1:** Demographic Characteristics,Variables associated with HO formation and Clinical Outcomes According to HO Formation Age is presented as mean±standard deviation (SD). Time from injury to surgery is presented as median (minimum-maximum). Student's t-test was used for age comparisons, Mann-Whitney U test for time from injury to surgery, and Fisher's exact test for categorical variables. Statistical significance was defined as p<0.05. HO: Heterotopic ossification. Table created by the authors using Microsoft Word (Microsoft, Redmond, WA).

Variable	Classification	HO formation				p*
		No (n=83)		Yes (n=11)		
		n	%	n	%	
Age at surgery (years)	Mean±SD (min-max)	83	35.7±7.8 (21-61)	11	36.7±10.7 (25-58)	0.692
Time from injury to surgery (days)	Median (min-max)	83	7 (2-10)	11	10 (7-30)	<0.001
Smoking	No	65	78.3%	10	90.9%	
	Yes	18	21.7%	1	9.1%	0.452
Anabolic steroid use	No	64	77.1%	2	18.2%	
	Yes	19	22.9%	9	81.8%	<0.001
Surgical side	Right	46	55.4%	5	45.5%	
	Left	37	44.6%	6	54.5%	0.749
Dominant side	No	25	30.1%	2	18.2%	
	Yes	58	69.9%	9	81.8%	0.503
Non-functional range of motion (ROM) at final follow-up	No	83	100.0%	9	81.8%	
	Yes	0	0.0%	2	18.2%	0.013
Required surgery	No	83	100.0%	9	81.8%	
	Yes	0	0.0%	2	18.2%	0.013

**Table 2:** Timing of Heterotopic Ossification Detection Following Distal Biceps Tendon Repair Values are presented as percentages of patients who developed heterotopic ossification (n=11). HO: heterotopic ossification. Table created by the authors using Microsoft Word (Microsoft, Redmond, WA).

Radiographic Follow-up	New HO Cases (n)	Percentage of HO Cases (%)
2 weeks	0	0.0
4 weeks	8	72.7
12 weeks	2	18.2
24 weeks	1	9.1

In univariate analyses, anabolic steroid use (81.8%; p<0.001) and a time interval greater than 10 days between injury and surgery (p<0.001) were significantly associated with the development of heterotopic ossification.

**Figure 1:**
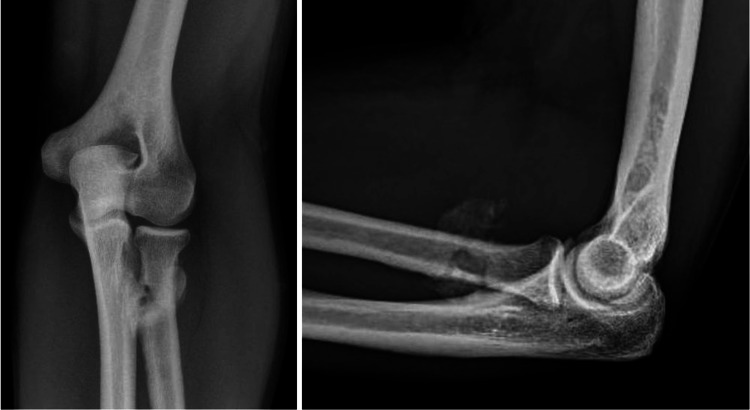
Anteroposterior and posterior X-ray view (author's original image) Heterotopic ossification identified six weeks after distal biceps tendon repair.

**Figure 2:**
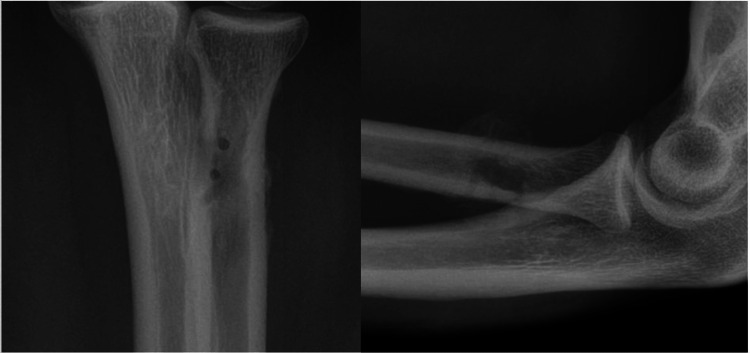
Anteroposterior and posterior X-ray view (author's original image) Spontaneous heterotopic ossification resorption at 24 weeks postoperatively.

Among the 11 patients who developed heterotopic ossification, six (54.5%) demonstrated spontaneous resorption within six months of follow-up. All patients with resorption achieved full recovery of range of motion at final follow-up, whereas those without resorption exhibited some degree of functional limitation. A representative case of heterotopic ossification identified at six weeks postoperatively is shown in Figure 1. Figure 2 demonstrates spontaneous radiographic resorption of heterotopic ossification during follow-up.

Functional outcomes assessed using the Mayo Elbow Performance Score (MEPS) demonstrated excellent results in most patients. Patients without heterotopic ossification achieved a mean MEPS of 97.8±2.5. Similarly, patients with spontaneous radiographic resorption of HO demonstrated comparable outcomes, with a mean MEPS of 94.2±2.0. Patients with persistent HO treated nonoperatively demonstrated good functional outcomes, with a mean MEPS of 83.3±2.9, while patients requiring surgical excision of symptomatic HO achieved a mean postoperative MEPS of 90.0±0 following reoperation and rehabilitation.

Among patients with limited range of motion, three demonstrated a functional arc according to Morrey criteria (30°-130° flexion-extension and 50°-50° pronation-supination) and did not undergo additional surgical treatment due to satisfactory clinical outcomes. In contrast, two patients (2.1% of the total cohort) presented with pain and non-functional range of motion and underwent surgical treatment consisting of heterotopic ossification excision and capsular release.

Postoperatively, prophylaxis with Celecoxib (200 mg twice daily for 14 days) was administered, combined with early rehabilitation, resulting in complete recovery of range of motion in both cases.

Overall, apart from heterotopic ossification, complications were observed in two patients (2.1%), including one case of transient neuropraxia of the lateral antebrachial cutaneous nerve and one case of partial wound dehiscence; both were managed nonoperatively and resolved without the need for additional surgical intervention.

## Discussion

The main finding of the present study was that the cumulative incidence of HO following distal biceps tendon repair using a double-incision technique was 11.7%, with only 2.1% of patients requiring surgical intervention. Furthermore, more than half of the cases (54.5%) demonstrated spontaneous radiographic resorption within six months, and all of these patients achieved full recovery of range of motion. These findings suggest that, although HO is relatively common, its clinical relevance may be limited in most cases.

Distal biceps tendon ruptures predominantly affect middle-aged men and are typically associated with the dominant limb, findings that are consistent with prior epidemiological studies [1,2]. The demographic profile observed in our cohort, with a predominance of male patients younger than 45 years and involvement of the dominant limb, aligns with the existing literature and reinforces the external validity of our sample.

The double-incision technique, originally described by Boyd and Anderson [3], remains a widely used approach due to its ability to restore the anatomic footprint of the distal biceps tendon. However, it has historically been associated with a higher incidence of HO when compared to single-incision techniques, as reported in several studies. The incidence of HO in our study (11.7%) falls within the lower range of previously reported values, which vary considerably across the literature depending on surgical technique, imaging protocols, and prophylactic strategies [4,5,7].

The pathophysiology of HO formation remains incompletely understood, but it is thought to involve a combination of local tissue trauma, periosteal disruption, and inflammatory signaling pathways that promote ectopic bone formation [6]. Despite this theoretical risk, our findings suggest that the majority of HO cases may follow a benign course.

A key contribution of the present study is the evaluation of the temporal evolution of HO. Most cases were identified early, predominantly at four weeks postoperatively, with no cases detected at two weeks, suggesting that routine early radiographic screening may have limited utility. Importantly, spontaneous resorption was observed in more than half of the affected patients, a finding that is rarely reported in the literature. Previous studies have described the prevalence and clinical implications of HO but have not extensively addressed its natural history or potential for resolution over time [7].

Another important finding was the identification of variables associated with HO development. The use of anabolic steroids and a delay greater than 10 days between injury and surgical repair were both significantly associated with the development of HO. The association with steroid use is consistent with prior reports suggesting that altered tendon biology and healing responses may predispose these patients to abnormal ossification [8]. Similarly, delayed surgical intervention may be associated with increased local inflammation and scar formation, potentially contributing to HO development.

From a clinical perspective, the most relevant finding is the low rate of symptomatic HO requiring intervention. Although 11.7% of patients developed radiographic HO, only 2.1% required surgical excision, and all achieved full recovery following reoperation.

These findings are supported by the favorable MEPS outcomes observed in most patients, including those treated nonoperatively, and are consistent with previous reports demonstrating that most cases of heterotopic ossification are asymptomatic or only minimally symptomatic. Together, these results suggest that many cases of HO may have limited clinical significance despite persistent radiographic findings [9]. This distinction between radiographic findings and clinical relevance is critical, as it may influence postoperative surveillance strategies and the indication for prophylactic measures.

The role of heterotopic ossification prophylaxis following distal biceps tendon repair remains controversial. Indomethacin has historically been the most commonly reported prophylactic regimen, typically administered at a dose of 75 mg daily for periods ranging from 10 days to six weeks postoperatively. In a recent systematic review focused specifically on distal biceps repair, Dave et al. found conflicting evidence regarding the effectiveness of indomethacin prophylaxis. While some studies reported a lower incidence of heterotopic ossification, others failed to demonstrate a significant benefit, leading the authors to conclude that current evidence is insufficient to support routine prophylaxis for all patients because of its limited efficacy and the potential adverse effects associated with prolonged NSAID use [12].

More recently, Ahmad et al. performed a systematic review and meta-analysis evaluating postoperative NSAID prophylaxis after elbow trauma surgery. Although neither indomethacin nor celecoxib demonstrated a statistically significant reduction in heterotopic ossification when analyzed separately, pooled analysis of all NSAID regimens showed a relative reduction of approximately 27% in the risk of postoperative heterotopic ossification compared with no prophylaxis. However, the authors emphasized the low quality of the available evidence and the need for additional prospective studies to confirm these findings [13].

Radiation therapy has also been described as a prophylactic option for heterotopic ossification, particularly following high-risk elbow and hip procedures. Nevertheless, the literature specific to distal biceps tendon repair remains scarce, and no comparative study evaluating radiotherapy for heterotopic ossification prevention after distal biceps repair was identified in the systematic review by Dave et al. [12]. Given the conflicting evidence regarding prophylactic efficacy and the low incidence of clinically significant heterotopic ossification observed in most published series, including the present study, routine prophylaxis remains controversial and should be individualized according to patient-specific risk factors.

The overall complication rate in our cohort was low, with only two minor complications (2.1%), including one transient neuropraxia of the lateral antebrachial cutaneous nerve and one case of partial wound dehiscence, both managed nonoperatively. These findings are in line with previously reported complication rates for distal biceps repair [14,15] and support the safety of the double-incision technique when performed by an experienced surgeon.

This study has several limitations. Its retrospective design introduces inherent risks of selection and information bias. Additionally, the absence of a control group treated with a single-incision technique limits direct comparison between surgical approaches. Radiographic assessment was limited to standard views, which may underestimate the true incidence of HO. Finally, although the sample size is relatively large for this condition, subgroup analyses may still be underpowered.

Despite these limitations, this study has important strengths, including a relatively large cohort, a standardized surgical technique performed by a single surgeon, and consistent radiographic follow-up. Moreover, the evaluation of the temporal evolution and potential resorption of HO represents a novel contribution to the literature.

## Conclusions

HO is a common finding after distal biceps tendon repair using the double-incision technique; however, many cases undergo spontaneous radiographic resorption and do not compromise functional outcomes. In contrast, persistent symptomatic HO may lead to functional limitations and occasionally require surgical excision. Conservative management appears appropriate for patients with spontaneous resorption or preserved function, whereas surgical intervention remains an effective option for those with persistent symptoms and restricted range of motion.
